# Mortality of individuals in a long-term cohort exposed to polybrominated biphenyls (PBBs)

**DOI:** 10.1186/s12940-025-01192-5

**Published:** 2025-07-01

**Authors:** Metrecia L. Terrell, Amila Adili, Robert B. Hood, Matthew P. Bursley, Hillary Barton, Melanie Pearson, Michele Marcus

**Affiliations:** 1 Department of Epidemiology, Emory University Rollins School of Public Health, 1518 Clifton Rd. NE, Atlanta, GA 30322 USA; 2 Division of Epidemiology, Ohio State University College of Public Health, 1841 Neil Avenue, Columbus, OH 43210 USA; 3 Michigan Department of Health and Human Services, Environmental Health Bureau, 333 South Grand Ave., 3rd Floor, Lansing, MI 48909 USA; 4 Gangarosa Department of Environmental Health, Emory University Rollins School of Public Health, 1518 Clifton Rd. NE, Atlanta, GA 30322 USA

**Keywords:** Polybrominated biphenyl, Brominated flame retardants, Persistent organic pollutants, Endocrine-disrupting chemicals, Long-term exposure, Mortality

## Abstract

**Background:**

This study is a long-term follow-up of individuals exposed to polybrominated biphenyls (PBBs). Widespread contamination of PBBs began in 1973 in Michigan when PBBs entered the food chain. PBBs are synthetic chemicals that were once used in industrial products. Their production in the United States ended following this incident. PBBs and other brominated flame retardants belong to a class of persistent organic pollutants that have been shown to affect human health. We conducted this study to investigate whether PBB exposure was associated with all-cause or cause-specific mortality risk.

**Methods:**

We included cohort data from 1976 (when the study began) and linked to National Death Index data obtained through the early release of 2021. Serum PBB concentrations were measured at enrollment in the study. We used survival analysis to estimate sex-specific hazard ratios (HR) and 95% confidence intervals (CI), adjusting for age and other important risk factors. The mortality study included 3,954 individuals.

**Results:**

In age-stratified analyses, higher PBB exposure was not associated with all-cause mortality risk in males or females. In cause-specific analyses conducted in the 16 or older group, we found no association between PBB exposure and circulatory system disease mortality. For all-cancer mortality, we found higher PBB exposure associated with increased risk of mortality in females (HR: 1.50, 95% CI: 1.02–2.22), which was inversed in males (HR: 0.68, 95% CI: 0.46–1.01). BMI appeared to modify the association between PBB exposure and all-cause mortality risk in males and all-cancer mortality risk in males and females.

**Conclusions:**

This comprehensive study found that the association between PBB exposure and cancer mortality risk varied by sex. Further research is needed to understand these sex-specific differences.

**Supplementary Information:**

The online version contains supplementary material available at 10.1186/s12940-025-01192-5.

## Background

Polybrominated biphenyls (PBBs) are brominated flame retardants (BFRs) that belong to a class of persistent organic pollutants (POPs) known to disrupt the endocrine system. United States production of PBB ended in the late 1970s following the PBB disaster, where millions of Michigan residents consumed food products contaminated with FireMaster, a mix of PBBs [1, 2]. The most abundant PBB congener, PBB-153, has been measured in serum samples for decades. PBBs remain a public health concern because of their lipophilic accumulation in the body, persistence in the environment, and documented long-term health effects. Furthermore, PBBs have been classified as a probable carcinogen to humans [3] and have half-life estimates that average approximately 12 years [4].

The Michigan Long-Term PBB Study was established 50 years ago after the PBB disaster. It was started by the Michigan Department of Public Health (now Michigan Department of Health and Human Services (MDHHS)), collaboratively with the U.S. Public Health Service, with support from multiple federal partners, to study the long-term health effects of exposure to PBB. Today, it is known as the Michigan PBB Registry, managed by Emory University, and includes some cohort members from the Michigan Long-Term PBB Study and additional PBB exposed individuals. Recently measured PBB-153 blood levels in this registry showed that 60% of individuals tested had levels higher than 95% of the U.S. population, according to the National Health and Nutrition Examination Survey (NHANES) [5]. Human studies have shown generational health effects among those directly exposed to PBB and those exposed *in utero* and through breastfeeding. Those directly exposed have shown increased risks of specific cancers, lymphoma, digestive system [6], and breast cancer [7], thyroid problems [8–10], and some autoimmune disorders [11]. The children of exposed mothers have shown adverse effects on some birth outcomes, growth and development [12–14], and reproductive outcomes [15]. The offspring of exposed fathers had lower birth weights with higher paternal exposure to PBB [16]. Further effects of PBB exposure on gene regulation [17], biological aging [18], and metabolic pathways [19, 20] have also been demonstrated.

There has only been one study of PBB exposure and adult mortality. This study was limited in both follow-up time (three years after the PBB disaster) and study design. It included workers from the former chemical plant and found that none of the workers who were regularly exposed to PBB died during follow-up [21]. Among other workers, too few mortality cases were reported at that time to support any meaningful findings. The only other study that examined PBB and mortality used NHANES data from 1999 to 2006 among participants aged 60 or older with background levels of exposure. The authors did not find that all-cause or cause-specific mortality increased with higher PBB exposure [22].

At community meetings held across Michigan, the affected community expressed concerns about a potential relationship between PBB and mortality. In response to this and to address a gap in the literature, we investigated whether PBB exposure was associated with an increased risk of all-cause or cause-specific mortality. This study includes 45 years of follow-up in PBB study participants and their mortality experience. The results of this study could advance policy interventions addressing persistent chemicals and inform clinical practice for earlier screenings of chronic diseases like cancer or cardiovascular disease.

## Methods

### Study population

The mortality study population includes individuals enrolled in the historic Michigan Long-Term PBB Study. During the initial enrollment period (1976–1978), MDHHS recruited several main groups: Michigan residents who either lived on farms quarantined by the State of Michigan, received food from these quarantined farms, or employees or household members of an employee at the Velsicol Chemical Corporation plant that produced PBB [23]. At enrollment, participants provided informed written consent and completed enrollment questionnaires about demographic information and their medical and exposure histories. Children enrolled in the study had questionnaires completed by their parents. Most participants also provided a serum sample for analysis of PBB exposure (~ 90%). Periodic updates of vital status, health, and exposure history continued over the years. The study was also expanded to include family members who did not initially enroll, as well as offspring born to participating families.

### Exposure assessment

During the initial enrollment period, participants provided a blood sample that was analyzed for PBB and PCB exposure by the MDHHS Bureau of Laboratories. Polychlorinated biphenyls (PCBs) were used as lubricants and coolants in electrical equipment until the late 1970s and are persistent in the environment [24]. Blood samples in this study were collected from non-fasting participants, and lipids were not measured. The analytical method for the enrollment PBB samples was based on the standard FireMaster FF-1 and quantitated using gas chromatography with electron capture detection [25]. FireMaster FF-1 was measured because it was the mixture involved in the PBB disaster. It is primarily congener PBB-153 (2,2’,4,4’,5,5’—Hexabromobiphenyl), making up over 50% of the mixture [26]. The detection limit (LOD) for serum PBB concentrations was 1 µg per liter (µg/L). The MDHHS Bureau of Laboratories also measured Aroclor 1254, a widely used commercial mixture of PCBs, using the double determination method [27]. The LOD of Aroclor 1254 was 5 µg/L.

### Vital status assessment

**Fig. 1:**
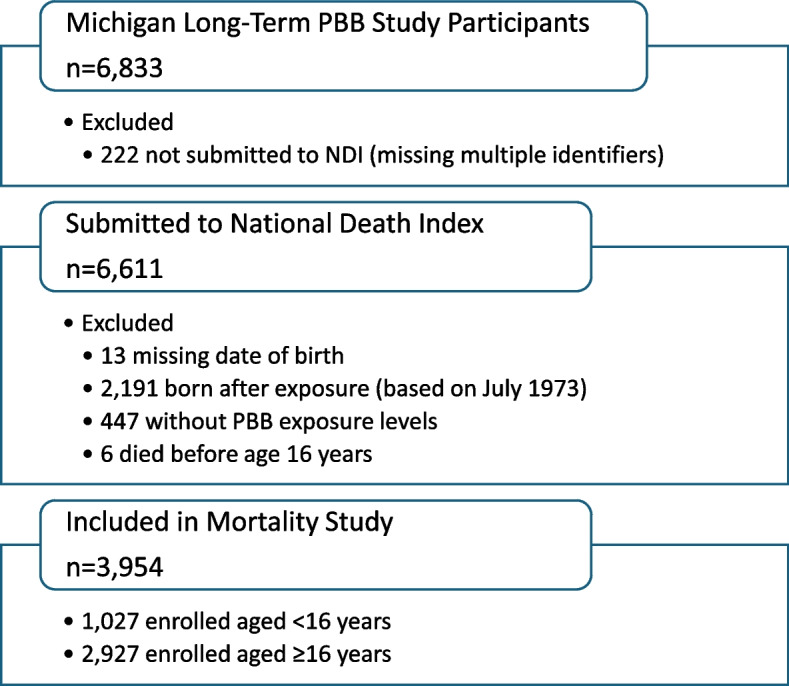
Flowchart of Michigan Long-Term PBB Study participants in the Mortality Study

Of the 6,833 individuals who had ever participated in the long-term study, data for 6,611 were submitted to the National Center for Health Statistics (NCHS) for linkage to the National Death Index (NDI) over the years of the cohort. For the mortality study, we included 3,954 participants. We excluded individuals who had incomplete dates of birth (*n* = 13), were born after the exposure incident began (exposed *in utero* and through breastfeeding, *n* = 2,191), did not have measured enrollment PBB exposure levels (*n* = 447), or died before age 16 (*n* = 6) (Fig. 1).

We excluded those born after the exposure incident began because most did not have blood samples collected for PBB exposure (*n* = 2,006 out of 2,191). The initial NDI submissions in the 1990s did not include the chemical workers or their family members because follow-up of this group had ended. This group was re-added in 2012 and included in subsequent NDI submissions in 2014 and 2022. The 2022 submission was linked to the NDI data covering 1979 through the 2021 early-release dataset. For these two later submissions, we included data for participants with unknown vital status or with known deceased status who did not have an electronic NDI record. We developed a matching protocol to review records in the returned NDI data files. Based upon a combination of matching identifiers (first and last name, social security number, and date of birth), a subsequent review of additional identifiers (sex, middle initial, and father’s surname, if available), and probabilistic scores and status codes, we classified records as true or false matches. We performed manual reviews in some instances to check data quality. Matches classified as “true” were then linked to the associated cause of death data files. We aggregated data over all submissions into a limited NDI match dataset, and causes of death were coded from the International Classification of Diseases (ICD) versions eight, nine, and ten. The underlying causes of death using ICD-8 and ICD-9 codes were recoded into corresponding ICD-10 codes. The MDHHS and Emory University Institutional Review Boards approved the study protocols.

### Statistical analysis

We constructed a combined dataset of the limited NDI match dataset with a covariate dataset for mortality analyses. The covariate dataset included age (at exposure and enrollment), sex, body mass index (BMI, calculated from self-reported height and weight at enrollment, categorized as underweight/normal weight: < 25 kg/m^2^, overweight: 25–29 kg/m^2^, and obese: ≥ 30 kg/m^2^), smoking history at enrollment (non-smoker, past smoker, current smoker), alcohol history at enrollment (nonuser, former/present user), exposure group, and enrollment serum PBB and PCB concentrations. We conducted age-stratified analyses because individuals enrolled before age 16 did not have information on adult height, weight, smoking history, or alcohol history, which we could consider in the analysis of those 16 or older. An exposure group was assigned during the study enrollment. Participants were classified as quarantined farm residents, recipients of food from quarantined farms, occupationally exposed chemical workers or their family members, and other participants who lived near the area of the Velsicol Chemical Plant, on non-quarantined farms, or who received food from non-quarantined farms. We considered analyses stratified by exposure group to account for differences in PBB exposure routes and PBB concentrations within the groups. The quarantined farm residents, chemical workers, and their family members may have experienced inhalation and dermal exposure to PBB, as well as ingesting it. Serum PBB and PCB concentrations were natural log-transformed, because of their skewed distributions, and non-detectable exposure concentrations (PBB, 13%; PCB, 27%) were imputed with the LOD/√2 prior to transformation. Age- and sex-specific exposure categories (low, moderate, and high) were created using the cumulative distribution of serum PBB and PCB concentrations.

We examined Kaplan–Meier curves and unadjusted hazard ratios (HR) to evaluate the risk of mortality by PBB exposure. Age was used as the time scale to allow for more precise adjustment for age [28, 29]. Participants entered the risk set when they enrolled in the study and remained until the date of death or the study cutoff date (December 2021), whichever occurred first. Study participants not identified as deceased were assumed to be alive as of the study cutoff date and contributed person-time to the analysis. We generated Cox regression models and estimated sex-specific HRs and 95% confidence intervals (CI) to satisfy model assumptions. The primary event of interest was all-cause mortality (ACM). Among those enrolled at age 16 or older, we considered cause-specific mortality (CSM) for the most common causes of death recorded, diseases of the circulatory system (females, 17.1%; males, 22.1%) and cancers (females, 10.8%; males, 11.3%). In the CSM models, participants who died from another cause were censored at their date of death. We also considered the potential confounders of BMI, smoking history, and alcohol history. Models were also stratified by birth cohort (< 1928, 1928–1947, ≥ 1948). Using likelihood ratio tests, we assessed multiplicative interactions (PBB x BMI, PBB x smoking history, and PBB x PCB) and considered effect modification where appropriate. All statistical analyses were performed using SAS/STAT software, Version 9.4 [30].

## Results

Participants who enrolled in the study younger than 16 years (*n* = 1,027) were exposed to PBB at a mean age of 6.8 years, and 4.7% died during follow-up (Table 1).

**Table 1:** Characteristics of Michigan Long-Term PBB Study participants (enrolled aged < 16 years)

Characteristic	Overall		Females (*n* = 498) N (%)	Males (*n* = 529) N (%)
	N	%		
Serum PBB concentrations^a^				
Low	448	43.6	244 (49.0)	204 (38.6)
Moderate	259	25.2	106 (21.3)	153 (28.9)
High	320	31.2	148 (29.7)	172 (32.5)
Serum PCB concentrations ^b^				
Low	424	49.3	230 (55.8)	194 (43.2)
Moderate	228	26.5	103 (25.0)	125 (27.8)
High	209	24.3	79 (19.2)	130 (29.0)
Missing	166			
Exposure Group				
Quarantined farm resident	528	51.4	251 (50.4)	277 (52.4)
Food recipient of quarantined farm	336	32.7	167 (33.5)	169 (32.0)
Family member of chemical worker	85	8.3	44 (8.8)	41 (7.8)
Other	78	7.6	36 (7.2)	42 (7.9)
Age at exposure (years)				
< 5	333	32.4	158 (31.7)	175 (33.1)
5–8	355	34.6	177 (35.5)	178 (33.7)
9–12	339	33.0	163 (32.7)	176 (33.3)
Age at enrollment (years)				
< 9	359	35.0	170 (34.1)	189 (35.7)
9–12	349	34.0	178 (35.7)	171 (32.3)
13–15	319	31.1	150 (30.1)	169 (32.0)
Vital Status				
Alive during follow-up	979	95.3	481 (96.6)	498 (94.1)
Died during follow-up	48	4.7	17 (3.4)	31 (5.9)

^a^ Serum PBB concentration categories: Females (low: ≤ 2 µg/L, moderate: 3–5 µg/L, high: ≥ 6 µg/L); Males (low: ≤ 3 µg/L, moderate: 4–7 µg/L, high: ≥ 8 µg/L)

^b^ Serum PCB concentration categories: (low: < 5 µg/L, moderate: 5–6 µg/L, high: > 6 µg/L)

Their median enrollment PBB concentration was 4 µg/L. There were slightly more males (52%) than females in this group, and the males had higher PBB concentrations than the females. Of those with PCB concentrations (*n* = 861), the median was 5 µg/L, the LOD. Males had slightly higher PCB levels than females. Participants in this group with higher PBB exposure had higher PCB exposure, were male, and were exposed to PBB when they were younger (Additional file 1).

Participants enrolled in the study when they were 16 years or older had a median enrollment PBB concentration of 3 µg/L. Like the younger group, males had higher concentrations than females. Their median enrollment PCB concentration was 7 µg/L. They were comprised of quarantined farm families (47%), followed by food recipients of quarantined farms (33%), and chemical workers and their family members (11%). Participants were, on average, 34 years old during the PBB exposure period. For covariates captured at enrollment, 47% were classified as overweight or obese, 42% were past or current smokers, and 65% were former or present users of alcohol. During follow-up, 48% died, more so for males (53%) than for females (43%) (Table 2).

**Table 2:** Characteristics of Michigan Long-Term PBB Study participants (enrolled aged ≥ 16 years)

Characteristic	Overall		Females (*n* = 1390) N (%)	Males (*n* = 1537) N (%)
	N	%		
Serum PBB concentrations ^a^				
Low	1008	34.4	572 (41.2)	436 (28.4)
Moderate	962	32.9	399 (28.7)	563 (36.6)
High	957	32.7	419 (30.1)	538 (35.0)
Serum PCB concentrations ^b^				
Low	706	26.9	314 (24.9)	392 (28.8)
Moderate	972	37.1	535 (42.4)	437 (32.1)
High	945	36.0	412 (32.7)	533 (39.1)
Missing	304			
Exposure Group				
Quarantined farm resident	1383	47.3	662 (47.6)	721 (46.9)
Food recipient of quarantined farm	966	33.0	499 (35.9)	467 (30.4)
Chemical worker or family member	319	10.9	111 (8.0)	208 (13.5)
Other	259	8.9	118 (8.5)	141 (9.2)
Age at exposure (years)				
10–15	412	14.1	190 (13.7)	222 (14.4)
16–35	1258	43.0	604 (43.5)	654 (42.6)
> 35	1257	42.9	596 (42.9)	661 (43.0)
Age at enrollment (years)				
16–24	797	27.2	381 (27.4)	416 (27.1)
25–35	667	22.8	323 (23.2)	344 (22.4)
36–50	757	25.9	366 (26.3)	391 (25.4)
> 50	706	24.1	320 (23.0)	386 (25.1)
Body Mass Index at enrollment				
Underweight/normal weight	1503	52.8	805 (59.5)	698 (46.7)
Overweight	970	34.1	349 (25.8)	621 (41.5)
Obese	374	13.1	198 (14.6)	176 (11.8)
Missing	80			
Smoking History at enrollment				
Non-smoker	1640	58.0	959 (71.5)	681 (45.8)
Past smoker	418	14.8	107 (8.0)	311 (20.9)
Current smoker	771	27.3	275 (20.5)	496 (33.3)
Missing	98			
Alcohol History at enrollment				
Nonuser	977	35.2	556 (42.3)	421 (28.8)
Former/present user	1801	64.8	758 (57.7)	1043 (71.2)
Missing	149			
Vital Status				
Alive during follow-up	1513	51.7	788 (56.7)	725 (47.2)
Died during follow-up	1414	48.3	602 (43.3)	812 (52.8)

^a^ Serum PBB concentration categories: Females (low: < 2 µg/L, moderate: 2–3 µg/L, high: ≥ 4 µg/L); Males (low: < 3 µg/L, moderate: 3–7 µg/L, high: ≥ 8 µg/L)

^b^ Serum PCB concentration categories: Females (low: < 5 µg/L, moderate: 5–7 µg/L, high: ≥ 8 µg/L); Males (low: < 6 µg/L, moderate: 6–8 µg/L, high: ≥ 9 µg/L)

Those with higher PBB exposure had higher PCB exposure and were male. Participants in the quarantined farm resident group and chemical worker or family member group were more likely to have higher PBB levels (Additional file 2, *p* < 0.001). For BMI, there was a significant inverse trend with the underweight/normal weight group more likely to have higher PBB levels than the obese group, who were more likely to have lower PBB levels (Additional file 2, *p* < 0.001).

For those in the study who enrolled before age 16, we did not find statistically significant differences in ACM risk with increasing PBB exposure in age-adjusted models. In females, the HR in the moderate PBB group was elevated (HR = 2.11, 95% CI: 0.76–5.81) but was not statistically significant with a wide confidence interval. The highest PBB group for females had an HR of 0.42 (95% CI: 0.09–1.99). Among males, the HRs were 0.75 (95% CI: 0.33–1.70) in the moderate PBB group and 0.44 (95% CI: 0.17–1.13) in the highest PBB group. Adjusting for PCB exposure or modeling PBB and PCB exposure as continuous variables did not substantially affect the results (Table 3).

**Table 3:** Association of serum PBB concentrations and all-cause mortality stratified by sex among Michigan Long-Term PBB Study participants (enrolled aged < 16 years)

	Females			Males		
Serum PBB concentrations ^ab^	N (%)	HR	95% CI	N (%)	HR	95% CI
Model 1:	498			529		
Low	244 (49.0)	1.00	Ref	204 (38.6)	1.00	Ref
Moderate	106 (21.3)	2.11	0.76–5.81	153 (28.9)	0.75	0.33–1.70
High	148 (29.7)	0.42	0.09–1.99	172 (32.5)	0.44	0.17–1.13
Model 2:	412			449		
Low	207 (50.2)	1.00	Ref	169 (37.6)	1.00	Ref
Moderate	87 (21.1)	1.71	0.55–5.26	132 (29.4)	0.84	0.34–2.05
High	118 (28.6)	0.47	0.10–2.20	148 (33.0)	0.38	0.12–1.18
Model 3:						
ln(PBB)	412	0.67	0.40–1.12	449	0.76	0.54–1.08

^a^ Serum PBB concentration categories: Females (low: ≤ 2 µg/L, moderate: 3–5 µg/L, high: ≥ 6 µg/L); Males (low: ≤ 3 µg/L, moderate: 4–7 µg/L, high: ≥ 8 µg/L); Serum PCB concentration categories: low: < 5 µg/L, moderate: 5–6 µg/L, high: > 6 µg/L

^b^ Model 1 adjusted for age; Model 2 adjusted for age and serum PCB concentration categories; Model 3 adjusted for age and ln(PCB)

In those enrolled when they were 16 or older, the HRs for PBB exposure and the association with ACM in age-adjusted models were not significant or elevated. For females in the highest PBB group, the HR was 1.03 (95% CI: 0.84–1.25), and for males, the HR was 0.95 (95% CI: 0.80–1.13). We observed similar results in models adjusted for PCB exposure or when exposures were continuous variables (Models 2–3, Table 4). Further, we did not find that stratification by birth cohort changed the model estimates.

**Table 4:** Association of serum PBB concentrations and all-cause mortality stratified by sex among Michigan Long-Term PBB Study participants (enrolled aged ≥ 16 years)

	Females			Males		
Serum PBB concentrations^ab^	N (%)	HR	95% CI	N (%)	HR	95% CI
Model 1:	1323			1466		
Low	543 (41.0)	1.00	Ref	413 (28.2)	1.00	Ref
Moderate	379 (28.7)	1.01	0.83–1.23	533 (36.4)	0.95	0.80–1.13
High	401 (30.3)	1.03	0.84–1.25	520 (35.5)	0.95	0.80–1.13
Model 2:	1201			1296		
Low	485 (40.4)	1.00	Ref	360 (27.8)	1.00	Ref
Moderate	349 (29.1)	1.04	0.85–1.28	470 (36.3)	0.92	0.76–1.10
High	367 (30.6)	1.08	0.87–1.33	466 (36.0)	0.93	0.77–1.12
Model 3:						
ln(PBB)	1201	1.00	0.94–1.07	1296	1.01	0.96–1.07

^a^ Serum PBB concentration categories: Females (low: < 2 µg/L, moderate: 2–3 µg/L, high: ≥ 4 µg/L); Males (low: < 3 µg/L, moderate: 3–7 µg/L, high: ≥ 8 µg/L); Serum PCB concentration categories: Females (low: < 5 µg/L, moderate: 5–7 µg/L, high: ≥ 8 µg/L); Males (low: < 6 µg/L, moderate: 6–8 µg/L, high: ≥ 9 µg/L)

^b^ Model 1 adjusted for age; Model 2 adjusted for age and serum PCB concentration categories; Model 3 adjusted for age and ln(PCB)

When we tested for interactions, the effect of PBB on ACM did not differ by smoking history or PCB exposure in the male or female models. However, the interaction term for PBB and BMI was significant in males (p-interaction = 0.01) but not in females (p-interaction = 0.12) when BMI was categorized (underweight/normal weight, overweight, and obese) (Additional file 3). Models stratified by BMI showed that in males, the risk of ACM increased in the overweight group among those that had higher PBB exposure (HR = 1.34, 95% CI: 1.03–1.73). However, the ACM risk was lower in the underweight/normal weight (HR = 0.71, 95% CI: 0.54–0.94) and the obese groups (HR = 0.64, 95% CI: 0.40–1.03). The male and female models showed insignificant interaction terms and attenuated hazard ratios with continuous BMI.

When we examined ACM by the exposure group variable, females who received food from quarantined farms or lived on quarantined farms had elevated hazard ratios. The HR for females who received food from quarantined farms was 1.29 (95% CI: 0.95–1.74), and among females who lived on quarantined farms was 1.37 (95% CI: 1.02–1.84) compared to the referent group that included those who lived near the area of the Velsicol Chemical Plant, on non-quarantined farms, or who received food from non-quarantined farms (Table 5). For males, the HRs for ACM trended lower than the referent group (quarantined farm resident, HR = 0.79, 95% CI: 0.61–1.00). In exposure group-stratified models, we did not find differences in ACM risk. Although based on small numbers in the chemical worker or family member group, the HRs were elevated but not statistically significant for females with higher PBB exposure (Model 4, Table 5).

**Table 5:** Association of exposure groups and all-cause mortality stratified by sex among Michigan Long-Term PBB Study participants (enrolled aged ≥ 16 years)

	Females			Males		
Models^a^	N (%)	HR	95% CI	N (%)	HR	95% CI
Model 1: Exposure groups	1323			1466		
Other	116 (8.8)	1.00	Ref	132 (9.0)	1.00	Ref
Food recipient of quarantined farm	484 (36.6)	1.29	0.95–1.74	449 (30.6)	0.85	0.66–1.10
Quarantined farm resident	638 (48.2)	1.37	1.02–1.84	698 (47.6)	0.79	0.61–1.00
Chemical worker or family member	85 (6.4)	1.22	0.76–1.96	187 (12.8)	0.96	0.71–1.30
Model 2 ^b^: Food recipient of quarantined farm	484			449		
Low	223 (46.1)	1.00	Ref	161 (35.9)	1.00	Ref
Moderate	127 (26.2)	0.96	0.69–1.35	145 (32.3)	0.84	0.62–1.14
High	134 (27.7)	0.95	0.68–1.32	143 (31.8)	1.11	0.81–1.51
Model 3^c^: Quarantined farm resident	638			698		
Low	224 (35.1)	1.00	Ref	243 (34.8)	1.00	Ref
Moderate	234 (36.7)	0.96	0.73–1.27	229 (32.8)	1.10	0.86–1.41
High	180 (28.2)	0.95	0.70–1.27	226 (32.4)	1.07	0.83–1.38
Model 4^d^: Chemical worker or family member	85			187		
Low	37 (43.5)	1.00	Ref	72 (38.5)	1.00	Ref
Moderate	25 (29.4)	1.34	0.54–3.33	51 (27.3)	0.79	0.48–1.29
High	23 (27.1)	1.61	0.59–4.38	64 (34.2)	0.86	0.54–1.38

^a^ Models adjusted for age

^b^ Food recipient of quarantined farm group serum PBB concentration categories: Females (low: < 2 µg/L, moderate: 2–3 µg/L, high: > 3 µg/L); Males (low: < 3 µg/L, moderate: 3–6 µg/L, high: > 6 µg/L)

^c^ Quarantined farm resident group serum PBB concentration categories: Females (low: ≤ 1 µg/L, moderate: 2–4 µg/L, high: > 4 µg/L); Males (low: ≤ 3 µg/L, moderate: 4–9 µg/L, high: > 9 µg/L)

^d^ Chemical worker or family member group serum PBB concentration categories: Females (low: < 2 µg/L, moderate: 2–3 µg/L, high: > 3 µg/L); Males (low: ≤ 4 µg/L, moderate: 5–17 µg/L, high: > 17 µg/L)

**Fig. 2:**
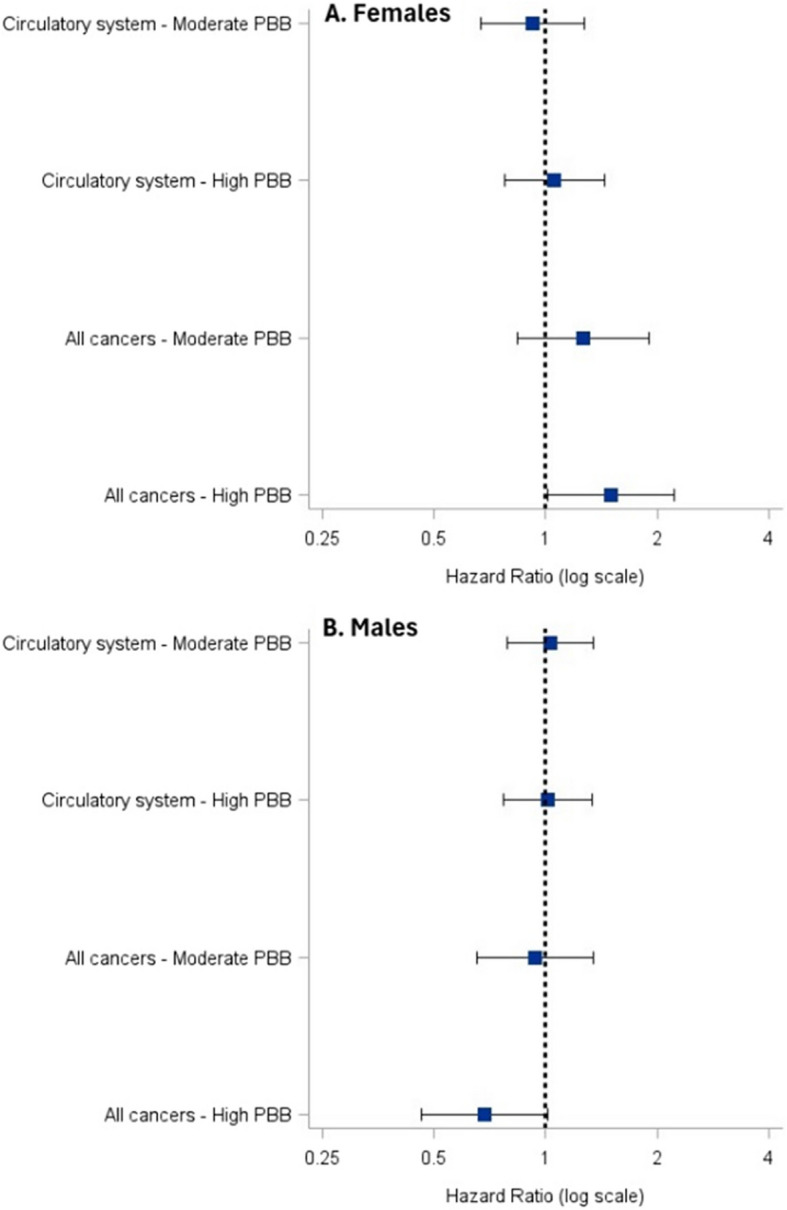
Association of serum PBB concentration categories (low, moderate, and high) and cause-specific mortality in **A**) Females and **B**) Males. Hazard ratios (lower risk: HR < 1; higher risk: HR > 1) and 95% CIs presented on the log-scale. Serum PBB concentration categories: Females (low: < 2 µg/L, moderate: 2–3 µg/L, high: ≥ 4 µg/L); Males (low: < 3 µg/L, moderate: 3–7 µg/L, high: ≥ 8 µg/L)

For cause-specific mortality (CSM) analyses, there was no association between PBB exposure and circulatory system disease mortality in the male or female models (Fig. 2). For all-cancer mortality, we found an increased risk with increasing PBB exposure in females. The HRs were 1.26 (95% CI: 0.84–1.90) and 1.50 (95% CI: 1.02–2.22) with moderate and high PBB exposure, respectively. Among males, the HRs were below 1.0 for all-cancer mortality risk (moderate PBB exposure, HR = 0.94, 95% CI: 0.65–1.34; high PBB exposure, HR = 0.68, 95% CI: 0.46–1.01) (Fig. 2). Although based on small numbers of deaths (5–6%), there was an increased risk of respiratory cancer mortality among females that had higher PBB exposure (HR = 3.82, 1.38–10.61). There was no association between PBB exposure and mortality because of digestive system cancers in males or females (Additional File 4).

The all-cancer mortality models revealed significant interactions between PBB exposure and BMI. Among the females, there was an increased risk of all-cancer mortality with continuous BMI (p-interaction = 0.04). However, among the males, there were lower risks in the underweight/normal and obese categories (p-interaction = 0.04, Additional File 5) with higher PBB exposure. There was no evidence of an interaction between PBB and BMI in the circulatory system disease mortality models (Additional File 6).

**Fig. 3:**
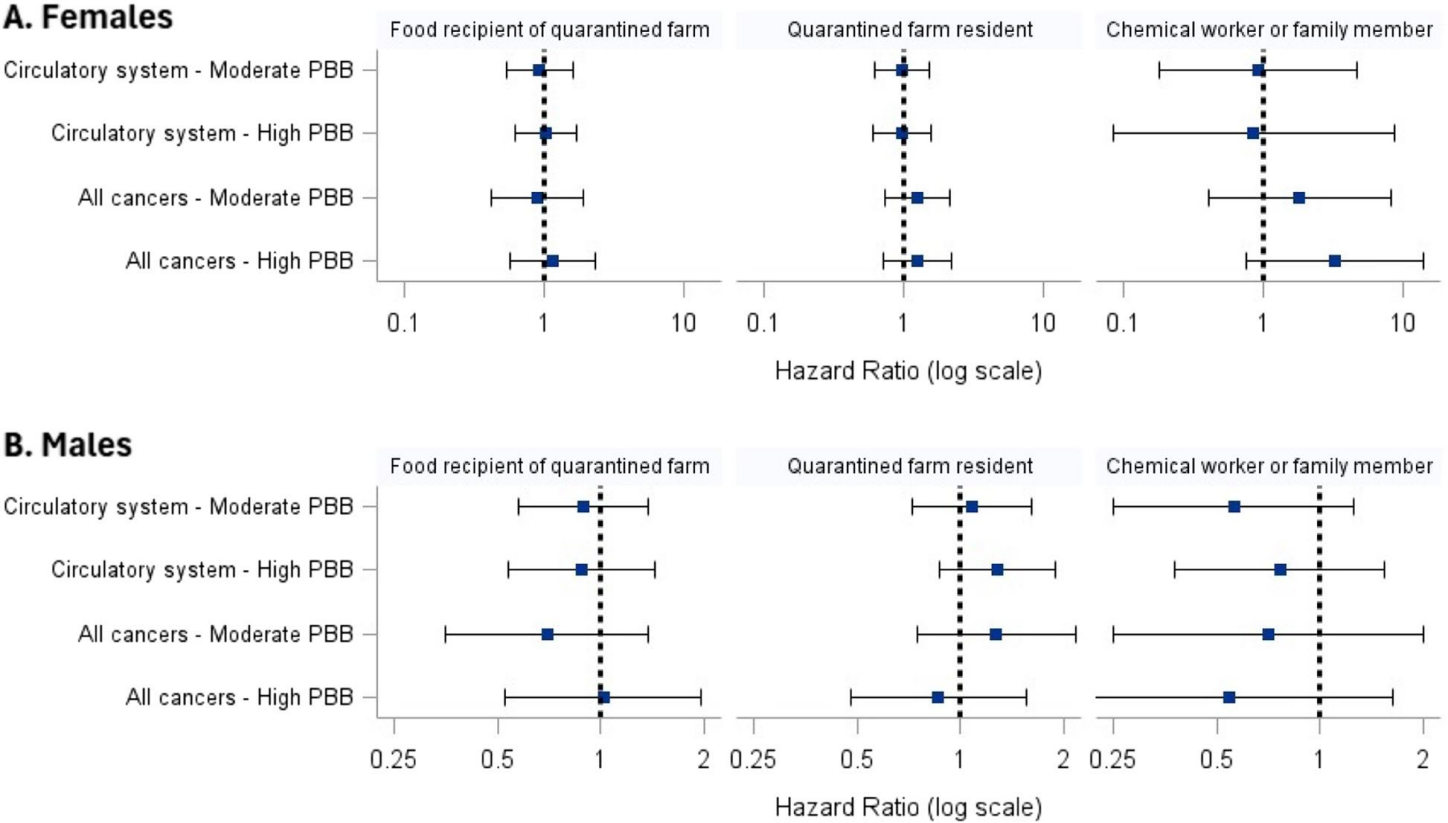
Association of serum PBB concentration categories (low, moderate, and high) and cause-specific mortality in **A**) Females and **B**) Males stratified by exposure group. Hazard ratios (lower risk: HR < 1; higher risk: HR > 1) and 95% CIs presented on the log-scale. Serum PBB concentration categories: Food recipient of quarantined farm group: Females (low: < 2 µg/L, moderate: 2–3 µg/L, high: > 3 µg/L); Males (low: < 3 µg/L, moderate: 3–6 µg/L, high: > 6 µg/L); Quarantined farm resident group: Females (low: ≤ 1 µg/L, moderate: 2–4 µg/L, high: > 4 µg/L); Males (low: ≤ 3 µg/L, moderate: 4–9 µg/L, high: > 9 µg/L); Chemical worker or family member group: Females (low: < 2 µg/L, moderate: 2–3 µg/L, high: > 3 µg/L); Males (low: ≤ 4 µg/L, moderate: 5–17 µg/L, high: > 17 µg/L)

We considered the association between PBB exposure and CSM risk by the exposure group variable. We found no statistically significant associations between increasing PBB exposure and risk of circulatory disease or all-cancer mortality across exposure groups (Fig. 3). In the female all-cancer mortality models, the HRs in the highest PBB exposure group showed a similar trend but were not statistically significant (food recipient of quarantined farm, HR = 1.17, 95% CI: 0.58–2.35; quarantined farm resident, HR = 1.26, 95% CI: 0.71–2.22; chemical worker or family member, HR = 3.31, 95% CI: 0.77–14.18).

## Discussion

This follow-up study of Michigan Long-Term PBB Study participants showed no increased all-cause mortality risk associated with PBB exposure in males or females. However, among males enrolled at age 16 or older, BMI appeared to modify this association. We conducted additional analyses for participants enrolled at age 16 or older. First, we considered the association between PBB exposure and cause-specific mortality. We found sex-specific differences in these analyses. In females, increased mortality risk from all cancers was associated with higher PBB exposure. For males, we found a slightly lower mortality risk from all cancers. The all-cancer mortality analyses also appeared to be modified by BMI for males and females. We found no association between PBB exposure and circulatory system disease mortality. Second, in models stratified by exposure group, we did not find higher PBB exposure associated with risk of all-cause mortality or cause-specific mortality in males or females.

While few studies of PBB exposure and mortality exist, the early study by Wong et al. [21], which included the Michigan chemical plant workers, did not find an excess of deaths among the workers. Further, the general U.S. population study of older adults in NHANES did not find an association between PBB-153 exposure and mortality [22].

Mortality studies of other persistent organic pollutants (POPs) with similar toxicological properties to PBBs have yielded inconsistent results. This may be because of differences in chemicals or congeners studied, populations (or comparison populations), or exposure routes, which makes results across studies difficult to compare. Studies that used specific PCB congeners or a summary measure of PCBs reported varied findings [31, 32]. The studies of electrical capacitor workers exposed to PCBs found no increase in all-cause mortality when comparing workers based on employment duration, hours worked, or to the U.S. general population [33, 34]. Studies of background levels of PCB exposure have shown no increase in all-cause mortality [22, 31, 32]. Research on two long-term cohorts exposed to PCBs and polychlorinated dibenzofurans (PCDFs) from contaminated cooking oil suggested varied results. These studies showed no difference in all-cause mortality [35] and an increase when compared to external populations [36, 37]. Several general population studies of polybrominated diphenyl ether (PBDE) exposure have not been associated with all-cause mortality [22, 32, 38].

Several studies and reviews have shown no association between POPs and cancer mortality [33, 37, 39, 40]. However, some studies have shown an overall association [38, 41], while others have only shown an association in sub-populations [34] or varying by sex [34–37]. Cardiovascular disease mortality studies have reported associations with PCB exposure [31, 32] and PCB and PCDF exposure [37] but not PBDE exposure [38].

While the underlying mechanism by which exposure to environmental contaminants, like PBB, could influence mortality risk is poorly understood, studies have shown these chemicals to have carcinogenic effects. In animal studies, PBBs have been shown to act as tumor promoters [42, 43]. Additionally, the role that these chemicals play in cancer progression has been suspected as a potential mechanism, particularly for breast cancer [44]. PBB and other endocrine disruptors can affect multiple organ systems, impacting normal hormone processes in the body. Endocrine-related effects have been demonstrated in this cohort [8–10], and an increase in several cancers has been associated with PBB exposure [6, 7].

All-cancer mortality risk was increased in females but not in males. This may be because of differences in sex hormones like estrogen, adiposity, and effects on hormone-sensitive organs. In an in vitro study, PBB-153 demonstrated estrogenic properties [45]. Because PBB accumulates in fat tissues, this could influence the rate at which PBB is eliminated from the body. PBB elimination studies have found a slower elimination rate with higher BMI [4, 46]. Also, slightly longer PBB half-life estimates have been found in females (13.8 years) than in males (11.2 years) [4]. In this cohort, sex-specific differences were found in an epigenetic study [47].

This study investigated potential interactions between PBB exposure and mortality risk factors. We did not find evidence of an interaction between PBB exposure and smoking history. We found that BMI influenced the association between PBB exposure and all-cause mortality risk in males and all-cancer mortality risk in both males and females. However, we lacked a direct measure of fat distribution or adiposity, making interpretation of these findings difficult. One study found an association between POPs and mortality only when considering fat mass [31]. Understanding the relationship between BMI and mortality is further complicated by its non-linear relationship [48–50]. Research suggests that the specific adipose tissue sites in which persistent organic pollutants are stored may influence both their elimination and long-term health effects [51–54].

The null results and lower risk estimates seen in some analyses could be because of our selected comparison group, which included participants with low levels of PBB exposure at enrollment. PBB levels in this group were probably still above background levels (LOD = 1 µg/L). We also attempted to investigate the association of PBB exposure and mortality risk by exposure route. The exposure group variable has been shown to be a predictor of current serum PBB levels. PBB levels were highest in the chemical workers, followed by quarantined farm residents, those who received food from quarantined farms, and family members of the chemical workers [5]. However, there may be misclassification that is likely non-differential and biased our results towards the null. The chemical worker group included their family members, and the quarantined farm resident group included some farmers who may have handled contaminated livestock feed. The cohort includes those occupationally exposed (primarily in the highest PBB exposure group) and therefore healthier than the general population. In addition, we used an internal comparison group to examine differences in mortality risk across levels of PBB exposure. Our reference group included participants with either non-detectable or low PBB levels. Since the referent group was not “unexposed” this may also bias the comparisons towards the null.

This study has several important strengths, the first being a long-term follow-up of this cohort. We determined the vital status of over 6,000 study participants and aggregated death certificate data from the NDI over many years. The exposure measurement used for the study was serum PBB levels measured in blood samples collected at enrollment (1976–1978), close to when levels were suspected to have been at their highest. In addition, we conducted sex-stratified analyses, which were essential to account for exposure levels and other inherent sex-specific differences.

There are also some limitations to mention. We were unable to include those born after the PBB exposure incident occurred. PBBs have been shown to cross the placenta and can be transferred via breastfeeding [55]. We used individual enrollment PBB levels in this study. However, in the group exposed to PBB *in utero* or through breastfeeding, most did not have a blood sample collected at enrollment (2,006 out of 2,191). Exposure during critical developmental periods has long-term health implications and is an area of research to be explored for mortality risk in this cohort.

Some cause-specific mortality analyses were subject to small numbers, resulting in hazard ratios with wide confidence intervals. These results should be interpreted with caution. We could not account for the elimination of PBB exposure or the change in exposure levels over time. This study included those exposed to higher levels of PBB, which may not reflect results expected in the general population with background levels of exposure. Further, we could not adjust for serum lipids because they were not measured in blood samples collected at enrollment in the late 1970s. While this may have resulted in some exposure misclassification, the serum PBB levels at enrollment likely reflected their long-term body burden. Within the first few years following the exposure incident, PBB levels in the blood and adipose tissue had reached equilibrium [56]. We could not account for other mortality risk factors, such as diet, exercise history or education level, which were not collected at enrollment into the cohort. Additionally, we did not have comparable Michigan mortality data; thus, we could not compare this cohort’s mortality experience to overall state rates.

Additional studies are needed to investigate the impact of PBB exposure on cancer incidence and survival as the cohort ages. Further research is needed for those born after the PBB incident began (e.g., using estimated *in utero* exposure levels). Their exposure to PBB occurred during vulnerable periods of development. In addition, study designs that can handle smaller sample sizes are essential to examine cause-specific mortality further, where we had small numbers.

## Conclusions

We conducted this study in response to the community’s concern about the long-term effects of PBB exposure on mortality. The results of this study will be shared with the PBB study participants and the PBB community partners. We provided some evidence that higher enrollment PBB levels did not negatively impact all-cause mortality risk. However, our results show some concern with the impact of PBB exposure on cancer mortality risk in females, which is consistent with earlier breast cancer studies in this cohort. PBB exposure has been associated with adverse health outcomes and remains a public health concern. It is important to continue to follow the life experiences of participants in this cohort.

## Supplementary Information

Additional file 1. Characteristics of Michigan Long-Term PBB Study participants by serum PBB concentrations (enrolled aged <16 years).

Additional file 2. Characteristics of Michigan Long-Term PBB Study participants by serum PBB concentrations (enrolled aged ≥16 years).

Additional file 3. Association of serum PBB concentration categories (low, moderate, and high) and risk of all-cause mortality stratified by sex and BMI among Michigan Long-Term PBB Study participants (enrolled aged ≥16 years).

Additional file 4. Association of serum PBB concentrations and cancer-specific mortality stratified by sex among Michigan Long-Term PBB Study participants.

Additional file 5. Association of serum PBB concentration categories (low, moderate, and high) and risk of all-cancer mortality stratified by sex and BMI among Michigan Long-Term PBB Study participants (enrolled aged ≥16 years).

Additional file 6. Association of serum PBB concentration categories (low, moderate, and high) and risk of circulatory system disease mortality stratified by sex and BMI among Michigan Long-Term PBB Study participants (enrolled aged ≥16 years).

## Data Availability

The data generated for this study is not publicly available and is restricted under a Data Use Agreement between Emory University and the Michigan Department of Health and Human Services. Contact the Corresponding Author about access to the data under the terms of this agreement.

## Abbreviations

PBB: Polybrominated biphenyl
HR: Hazard ratio
CI: Confidence interval
BFR: Brominated flame retardant
POP: Persistent organic pollutant
MDHHS: Michigan Department of Health and Human Services
PBDE: Polybrominated diphenyl ethers
NHANES: National Health and Nutrition Examination Survey
PCB: Polychlorinated biphenyl
LOD: Limit of detection
NCHS: National Center for Health Statistics
NDI: National Death Index
ICD: International Classification of Diseases
BMI: Body mass index
ACM: All-cause mortality
CSM: Cause-specific mortality
PCDF: Polychlorinated dibenzofuran
