# Supplementary material for: Mortality of individuals in a long-term cohort exposed to polybrominated biphenyls (PBBs)
Source: Environ Health. 2025 Jul 1;24:42. doi: 10.1186/s12940-025-01192-5 (PMC12219131; doi:10.1186/s12940-025-01192-5)
Supplement: Supplementary file 1 — Additional file 1. Characteristics of Michigan Long-Term PBB Study participants by serum PBB concentrations (enrolled aged <16 years). [file 12940_2025_1192_MOESM1_ESM.docx]

**Additional file 1.** Characteristics of Michigan Long-Term PBB Study participants by serum PBB concentrations (enrolled aged <16 years)

|  | Overall | | | | Serum PBB concentrations ^a^ | | | | | | |
| --- | --- | --- | --- | --- | --- | --- | --- | --- | --- | --- | --- |
| Characteristic | N | | % | | Low (n=448)  N (%) | | Moderate (n=259)  N (%) | | High (n=320)  N (%) | | p-value ^c^ |
| Serum PCB concentrations ^b^ |  | |  | |  | |  | |  | | 0.035 |
| Low | 424 | | 49.2 | | 204 (54.3) | | 99 (45.2) | | 121 (45.5) | |  |
| Moderate | 228 | | 26.5 | | 87 (23.1) | | 71 (32.4) | | 70 (26.3) | |  |
| High | 209 | | 24.3 | | 85 (22.6) | | 49 (22.4) | | 75 (28.2) | |  |
| Missing | 166 | |  | |  | |  | |  | |  |
| Sex |  | |  | |  | |  | |  | | 0.002 |
| Female | 498 | | 48.5 | | 244 (54.5) | | 106 (40.9) | | 148 (46.3) | |  |
| Male | 529 | | 51.5 | | 204 (45.5) | | 153 (59.1) | | 172 (53.8) | |  |
| Exposure Group | |  | |  | |  | |  | |  | <0.001 |
| Quarantined farm resident | 528 | | 51.4 | | 199 (44.4) | | 137 (52.9) | | 192 (60.0) | |  |
| Food recipient of quarantined farm | 336 | | 32.7 | | 172 (38.4) | | 73 (28.2) | | 91 (28.4) | |  |
| Family member of chemical worker | 85 | | 8.3 | | 35 (7.8) | | 23 (8.9) | | 27 (8.4) | |  |
| Other | 78 | | 7.6 | | 42 (9.4) | | 26 (10.0) | | 10 (3.1) | |  |
| Age at exposure |  | |  | |  | |  | |  | | 0.010 |
| <5 | 333 | | 32.4 | | 120 (26.8) | | 94 (36.3) | | 119 (37.2) | |  |
| 5-8 | 355 | | 34.6 | | 176 (39.3) | | 80 (30.9) | | 99 (30.9) | |  |
| 9-12 | 339 | | 33.0 | | 152 (33.9) | | 85 (32.8) | | 102 (31.9) | |  |
| Age at enrollment |  | |  | |  | |  | |  | | 0.005 |
| <9 | 359 | | 35.0 | | 128 (28.6) | | 102 (39.4) | | 129 (40.3) | |  |
| 9-12 | 349 | | 34.0 | | 171 (38.2) | | 78 (30.1) | | 100 (31.3) | |  |
| 13-15 | 319 | | 31.1 | | 149 (33.3) | | 79 (30.5) | | 91 (28.4) | |  |
| Vital Status |  | |  | |  | |  | |  | | 0.075 |
| Alive during follow-up | 979 | | 95.3 | | 424 (94.6) | | 243 (93.8) | | 312 (97.5) | |  |
| Died during follow-up | 48 | | 4.7 | | 24 (5.4) | | 16 (6.2) | | 8 (2.5) | |  |

^a^ Serum PBB concentration categories: Females (low: ≤2 µg/L, moderate: 3-5 µg/L, high: ≥6 µg/L); Males (low: ≤3 µg/L, moderate: 4-7 µg/L, high: ≥8 µg/L)

^b^ Serum PCB concentration categories: (low: <5 µg/L, moderate: 5-6 µg/L, high: >6 µg/L)

^c^ Pearson chi-square p-values
