# Supplementary material for: Mortality of individuals in a long-term cohort exposed to polybrominated biphenyls (PBBs)
Source: Environ Health. 2025 Jul 1;24:42. doi: 10.1186/s12940-025-01192-5 (PMC12219131; doi:10.1186/s12940-025-01192-5)
Supplement: Supplementary file 2 — Additional file 2. Characteristics of Michigan Long-Term PBB Study participants by serum PBB concentrations (enrolled aged ≥16 years). [file 12940_2025_1192_MOESM2_ESM.docx]

**Additional file 2.** Characteristics of Michigan Long-Term PBB Study participants by serum PBB concentrations (enrolled aged ≥16 years)

|  | Overall | | Serum PBB concentrations ^a^ | | | |
| --- | --- | --- | --- | --- | --- | --- |
| Characteristic | N | % | Low (n=1008)  N (%) | Moderate (n=962)  N (%) | High (n=957)  N (%) | p-value ^c^ |
| Serum PCB concentrations ^b^ |  |  |  |  |  | <0.001 |
| Low | 706 | 26.9 | 278 (31.2) | 232 (26.8) | 196 (22.7) |  |
| Moderate | 972 | 37.1 | 342 (38.3) | 343 (39.6) | 287 (33.2) |  |
| High | 945 | 36.0 | 272 (30.5) | 291 (33.6) | 382 (44.2) |  |
| Missing | 304 |  |  |  |  |  |
| Sex |  |  |  |  |  | <0.001 |
| Female | 1390 | 47.5 | 572 (56.8) | 399 (41.5) | 419 (43.8) |  |
| Male | 1537 | 52.5 | 436 (43.3) | 563 (58.5) | 538 (56.2) |  |
| Exposure Group |  |  |  |  |  | <0.001 |
| Quarantined Farm Resident | 1383 | 47.3 | 403 (40.0) | 453 (47.1) | 527 (55.1) |  |
| Food recipient of quarantined farm | 966 | 33.0 | 399 (39.6) | 299 (31.1) | 268 (28.0) |  |
| Chemical worker or family member | 319 | 10.9 | 99 (9.8) | 93 (9.7) | 127 (13.3) |  |
| Other | 259 | 8.9 | 107 (10.6) | 117 (12.6) | 35 (3.7) |  |
| Age at exposure |  |  |  |  |  | 0.448 |
| 10-15 | 412 | 14.1 | 150 (14.9) | 128 (13.3) | 134 (14.0) |  |
| 16-35 | 1258 | 43.0 | 425 (42.2) | 403 (41.9) | 430 (44.9) |  |
| >35 | 1257 | 42.9 | 433 (43.0) | 431 (44.8) | 393 (41.1) |  |
| Age at enrollment |  |  |  |  |  | 0.875 |
| 16-24 | 797 | 27.2 | 280 (27.8) | 250 (26.0) | 267 (27.9) |  |
| 25-35 | 667 | 22.8 | 229 (22.7) | 215 (22.4) | 223 (23.3) |  |
| 36-50 | 757 | 25.9 | 265 (26.3) | 253 (26.3) | 239 (25.0) |  |
| >50 | 706 | 24.1 | 234 (23.2) | 244 (25.4) | 228 (23.8) |  |
| Body Mass Index at enrollment |  |  |  |  |  | <0.001 |
| Underweight/normal weight | 1503 | 52.8 | 465 (47.5) | 493 (53.0) | 545 (58.2) |  |
| Overweight | 970 | 34.1 | 356 (36.4) | 310 (33.3) | 304 (32.4) |  |
| Obese | 374 | 13.1 | 158 (16.1) | 128 (13.8) | 88 (9.4) |  |
| Missing | 80 |  |  |  |  |  |
| Smoking History at enrollment |  |  |  |  |  | 0.630 |
| Non-smoker | 1640 | 58.0 | 576 (59.6) | 532 (57.3) | 532 (57.0) |  |
| Past smoker | 418 | 14.8 | 137 (14.2) | 146 (15.7) | 135 (14.5) |  |
| Current smoker | 771 | 27.3 | 253 (26.2) | 251 (27.0) | 267 (28.6) |  |
| Missing | 98 |  |  |  |  |  |
| Alcohol History at enrollment |  |  |  |  |  | 0.997 |
| Nonuser | 977 | 35.2 | 333 (35.1) | 319 (35.2) | 325 (35.3) |  |
| Former/present user | 1801 | 64.8 | 616 (64.9) | 588 (64.8) | 597 (64.8) |  |
| Missing | 149 |  |  |  |  |  |
| Vital Status |  |  |  |  |  | 0.620 |
| Alive during follow-up | 1513 | 51.7 | 529 (52.5) | 485 (50.4) | 499 (52.1) |  |
| Died during follow-up | 1414 | 48.3 | 479 (47.5) | 477 (49.6) | 458 (47.9) |  |

^a^ Serum PBB concentration categories: Females (low: <2 µg/L, moderate: 2-3 µg/L, high: ≥4 µg/L);

Males (low: <3 µg/L, moderate: 3-7 µg/L, high: ≥8 µg/L)

^b^ Serum PCB concentration categories: Females (low: <5 µg/L, moderate: 5-7 µg/L, high: ≥8 µg/L);

Males (low: <6 µg/L, moderate: 6-8 µg/L, high: ≥9 µg/L)

^c^ Pearson chi-square p-values
