# Supplementary material for: Mortality of individuals in a long-term cohort exposed to polybrominated biphenyls (PBBs)
Source: Environ Health. 2025 Jul 1;24:42. doi: 10.1186/s12940-025-01192-5 (PMC12219131; doi:10.1186/s12940-025-01192-5)
Supplement: Supplementary file 4 — Additional file 4. Association of serum PBB concentrations and cancer-specific mortality stratified by sex among Michigan Long-Term PBB Study participants. [file 12940_2025_1192_MOESM4_ESM.docx]

**Additional File 4.** Association of serum PBB concentrations and cancer-specific mortality stratified by sex among Michigan Long-Term PBB Study participants.

|  | Females | | | Males | | |
| --- | --- | --- | --- | --- | --- | --- |
| Cancer Type ^a^ | N (%) | HR | 95% CI | N (%) | HR | 95% CI |
| Digestive Cancer | 1323 |  |  | 1466 |  |  |
| Low | 543 (41.0) | 1.00 | Ref | 413 (28.2) | 1.00 | Ref |
| Moderate | 379 (28.7) | 1.21 | 0.60-2.42 | 533 (36.4) | 0.94 | 0.45-1.95 |
| High | 401 (30.3) | 0.72 | 0.32-1.62 | 520 (35.5) | 0.61 | 0.27-1.39 |
| Respiratory Cancer | 1323 |  |  | 1466 |  |  |
| Low | 543 (41.0) | 1.00 | Ref | 413 (28.2) | 1.00 | Ref |
| Moderate | 379 (28.7) | 1.65 | 0.50-5.40 | 533 (36.4) | 0.88 | 0.43-1.81 |
| High | 401 (30.3) | 3.82 | 1.38-10.61 | 520 (35.5) | 0.64 | 0.29-1.42 |

^a^ Models adjusted for age; Serum PBB concentration categories (low, moderate, high): Females (low: <2 µg/L, moderate: 2-3 µg/L, high: ≥4 µg/L); Males (low: <3 µg/L, moderate: 3-7 µg/L, high: ≥8 µg/L)
