# Supplementary material for: Mortality of individuals in a long-term cohort exposed to polybrominated biphenyls (PBBs)
Source: Environ Health. 2025 Jul 1;24:42. doi: 10.1186/s12940-025-01192-5 (PMC12219131; doi:10.1186/s12940-025-01192-5)
Supplement: Supplementary file 5 — Additional file 5. Association of serum PBB concentration categories (low, moderate, and high) and risk of all-cancer mortality stratified by sex and BMI among Michigan Long-Term PBB Study participants (enrolled aged ≥16 years). [file 12940_2025_1192_MOESM5_ESM.docx]

**Additional file 5.** Association of serum PBB concentration categories (low, moderate, and high) and risk of all-cancer mortality stratified by sex and BMI among Michigan Long-Term PBB Study participants (enrolled aged ≥16 years)

|  | Females  PBB x BMI | | | | Males  PBB x BMI | | | |
| --- | --- | --- | --- | --- | --- | --- | --- | --- |
| Models with serum PBB concentration categories ^a^ | N | HR | 95% CI | p-interaction | N | HR | 95% CI | p-interaction |
| BMI categorized | 1323 |  |  | 0.18 | 1466 |  |  | 0.04 |
| Underweight/normal weight |  |  |  |  |  |  |  |  |
| Low | 294 | 1.00 | Ref |  | 157 | 1.00 | Ref |  |
| Moderate | 231 | 1.90 | 1.03-3.50 |  | 246 | 0.62 | 0.36-1.09 |  |
| High | 259 | 2.23 | 1.24-4.01 |  | 273 | 0.48 | 0.27-0.86 |  |
| Overweight |  |  |  |  |  |  |  |  |
| Low | 157 | 1.00 | Ref |  | 193 | 1.00 | Ref |  |
| Moderate | 91 | 1.06 | 0.50-2.27 |  | 216 | 1.74 | 0.97-3.11 |  |
| High | 98 | 1.45 | 0.72-2.90 |  | 206 | 1.15 | 0.60-2.19 |  |
| Obese |  |  |  |  |  |  |  |  |
| Low | 92 | 1.00 | Ref |  | 63 | 1.00 | Ref |  |
| Moderate | 57 | 0.67 | 0.27-1.65 |  | 71 | 0.39 | 0.16-0.95 |  |
| High | 44 | 0.59 | 0.21-1.64 |  | 41 | 0.37 | 0.13-1.06 |  |
| BMI continuous ^b^ |  |  |  | 0.04 |  |  |  | 0.82 |
| Low | 543 | 1.00 | Ref |  | 413 | 1.00 | Ref |  |
| Moderate | 379 | 1.45 | 0.94-2.23 |  | 533 | 0.92 | 0.64-1.33 |  |
| High | 401 | 1.71 | 1.13-2.58 |  | 520 | 0.68 | 0.46-1.01 |  |

^a^ Models adjusted for age; Serum PBB concentration categories: Females (low: <2 µg/L, moderate: 2-3 µg/L, high: ≥4 µg/L); Males (low: <3 µg/L, moderate: 3-7 µg/L, high: ≥8 µg/L)

^b^ BMI continuous model estimated at an average BMI of 24.8 kg/m^2^ for females and 25.6 kg/m^2^ for males
