# Supplementary material for: Mortality of individuals in a long-term cohort exposed to polybrominated biphenyls (PBBs)
Source: Environ Health. 2025 Jul 1;24:42. doi: 10.1186/s12940-025-01192-5 (PMC12219131; doi:10.1186/s12940-025-01192-5)
Supplement: Supplementary file 6 — Additional file 6. Association of serum PBB concentration categories (low, moderate, and high) and risk of circulatory system disease mortality stratified by sex and BMI among Michigan Long-Term PBB Study participants (enrolled aged ≥16 years). [file 12940_2025_1192_MOESM6_ESM.docx]

**Additional File 6** Association of serum PBB concentration categories (low, moderate, and high) and risk of circulatory system disease mortality stratified by sex and BMI among Michigan Long-Term PBB Study participants (enrolled aged ≥16 years)

|  | Females  PBB x BMI | | | | Males  PBB x BMI | | | |
| --- | --- | --- | --- | --- | --- | --- | --- | --- |
| Models with serum PBB concentration categories ^a^ | N | HR | 95% CI | p-interaction | N | HR | 95% CI | p-interaction |
| BMI categorized | 1323 |  |  | 0.67 | 1466 |  |  | 0.25 |
| Underweight/normal weight |  |  |  |  |  |  |  |  |
| Low | 294 | 1.00 | Ref |  | 157 | 1.00 | Ref |  |
| Moderate | 231 | 1.03 | 0.61-1.75 |  | 246 | 0.69 | 0.42-1.12 |  |
| High | 259 | 1.17 | 0.71-1.91 |  | 273 | 0.74 | 0.46-1.18 |  |
| Overweight |  |  |  |  |  |  |  |  |
| Low | 157 | 1.00 | Ref |  | 193 | 1.00 | Ref |  |
| Moderate | 91 | 0.99 | 0.58-1.70 |  | 216 | 1.18 | 0.81-1.72 |  |
| High | 98 | 1.25 | 0.73-2.15 |  | 206 | 1.32 | 0.90-1.93 |  |
| Obese |  |  |  |  |  |  |  |  |
| Low | 92 | 1.00 | Ref |  | 63 | 1.00 | Ref |  |
| Moderate | 57 | 0.76 | 0.41-1.41 |  | 71 | 1.28 | 0.67-2.44 |  |
| High | 44 | 0.81 | 0.43-1.50 |  | 41 | 0.99 | 0.47-2.06 |  |
| BMI continuous ^b^ |  |  |  | 0.63 |  |  |  | 0.56 |
| Low | 543 | 1.00 | Ref |  | 413 | 1.00 | Ref |  |
| Moderate | 379 | 0.97 | 0.69-1.38 |  | 533 | 0.97 | 0.72-1.29 |  |
| High | 401 | 1.14 | 0.82-1.60 |  | 520 | 1.04 | 0.78-1.38 |  |

^a^ Models adjusted for age; Serum PBB concentration categories: Females (low: <2 µg/L, moderate: 2-3 µg/L, high: ≥4 µg/L); Males (low: <3 µg/L, moderate: 3-7 µg/L, high: ≥8 µg/L)

^b^ BMI continuous model estimates at an average BMI of 24.8 kg/m^2^ for females and 25.6 kg/m^2^ for males
